# Editorial: The predictive benefits of inflammatory markers in cancers of the liver

**DOI:** 10.3389/fonc.2024.1386388

**Published:** 2024-03-15

**Authors:** Gianluca Rompianesi, Domenico Tamburrino

**Affiliations:** ^1^ Hepato-Bilio-Pancreatic, Minimally-invasive, Robotic and Transplant Surgery Unit, Federico II University Hospital, Naples, Italy; ^2^ Division of Pancreatic and Transplant Surgery, Pancreas Translational & Clinical Research Center, San Raffaele Scientific Institute, Milan, Italy

**Keywords:** liver cancer, inflammatory markers, hepatocellular carcinoma (HCC), cholangiocarcinoma (CCA), predictive ability

Primary malignant liver tumours still represent an intricated scenario that challenges clinicians in their strive for achieving prompt and accurate diagnosis, effective therapies and treatments, and the best possible outcomes and quality of life. The frequency and mortality rates of liver tumours mandate a constant effort to improve the tools that can assist physician and surgeons in their clinical endeavours. Biomarkers have shown the potential to be a powerful complimentary tool to optimize patient outcomes by improving diagnosis, prognosis, and treatment response prediction. It is well known that inflammation is a hallmark of cancer, contributing to several aspects of tumour development and progression as well as to the response to therapy. Therefore, inflammatory biomarkers could play a key role in all stages of cancer treatment.

This Research Topic focuses on the various roles and benefits of inflammatory markers in liver cancers.

Hepatocellular carcinoma (HCC) is the most common primary liver cancer and is still burdened by high recurrence rates and mortality. Its management is still a matter of debate, and possible therapeutic strategies range from locoregional treatments to immunotherapy and surgery, including liver transplantation. In order to identify the best approach, several factors have to be taken into consideration, including the patient’s general conditions, liver disease status, and tumour stage, all included in decision-making algorithms and the recently introduced concepts of therapeutic hierarchy (1, 2). A key factor allowing satisfactory long-term outcomes is accurate pre-intervention prognostication that would assist clinicians in allocating patients to the best possible treatment with a more reliable and personalised approach. In recent years, a strong correlation between systemic inflammation and HCC prognosis has been described, with several systemic and pathological markers associated with survival and recurrence.

Giannone et al. in their review describe the features of the inflammatory microenvironment in all stages of HCC carcinogenesis, with special focuses on serum markers and gene signatures and their ability in predicting HCC recurrence and survival.

The surgical approach represents one of the preferred treatments in early-stage HCC, but in the context of liver cirrhosis, it can be complex and burdened by a high incidence of postoperative complications. Among them, one of the most feared is the post-hepatectomy liver failure (PHLF). Among the inflammatory indexes, AST-to-platelet ratio index (APRI) has the characteristic of reflecting the progression of liver cirrhosis, thanks to the increased AST release consequent to cell damage, and the severity of portal hypertension, represented by a a decrease in circulating platelets values. APRI has been investigated by Fang et al., who retrospectively collected a sample of 488 HCC patients undergoing liver resection, and included it in a nomogram that outperformed the MELD, ALPI and CP scores in predicting PHLF (C-index of 0.845, 95%CI, 0.806-0.884).

In case of single, large (>5 cm) HCC, the recurrence rates are high, even after radical surgery. To optimise outcomes, a two-step approach including Transcatheter Arterial Embolization (TACE) before liver resection has been explored, but heterogeneous results have been observed and no clear oncological benefit demonstrated. Zhang et al. evaluated the presence of circulating tumour cells (CTCs) in patients with large HCC, and found that only in CTC-positive patients, preoperative TACE reduced early recurrence and improved long-term survival, allowing better patient selection and treatment allocation.

The high recurrence rates after HCC resection mandate strict follow-up and frequently, patients need further treatments, including re-do resections, locoregional treatments and salvage liver transplantation. Chen and Wang in their retrospective analysis on 896 HCC-HBV patients identified pre-operative IL-25 levels as predictor of postoperative overall and recurrence-free survival. Patients with IL-25 levels <14.9 μg/ml had significantly better outcomes, representing a valuable diagnostic and prognostic tool, especially in cases of alfa-fetoprotein-negative HCC. In a similar population of patients, Wenpei et al. constructed a combined inflammation and pathology model (CIP) to investigate the predictive value of preoperative neutrophil-to-lymphocyte ratio (NLR), platelet-to-lymphocyte ratio (PLR), systemic inflammation response index (SIRI), and systemic immune inflammation index (SII) for early recurrence in HCC-HBV patients undergoing liver resection. The CIP model showed a good predictive ability, with an AUC of 0.804.

Several international guidelines identify TACE as the one of the principal treatment options for patients unresectable HCC. Although TACE can potentially be repeated in case of incomplete treatment or recurrence, reduced efficacy and refractoriness can be observed. Identifying patients experiencing TACE refractoriness would have great benefits, as the early use of combination therapy confers significant survival advantages. Xia et al. described how high plasma arginase-1 (ARG1) expression was independently associated with a lower incidence of early TACE refractoriness and constructed a nomogram also including tumour size and number and platelet count, predicting refractoriness with an AUC of 0.833 (95%CI 0.791-0.875).

Patients with unresectable HCC often present with a large tumour size, as well as vascular invasion or distant metastases. In these cases, the ability of TACE to achieve complete tumour necrosis is limited and could paradoxically contribute to tumour recurrence and dissemination through increased expression of programmed cell death ligand 1 (PD-L1) and vascular endothelial growth factors (VEGF). Therefore, treatments with immune checkpoint inhibitors (ICIs) and tyrosine kinase inhibitors (TKIs) can be indicated in unresectable HCC in combination with TACE. Guo et al. retrospectively evaluated 98 patients undergoing TACE+ICIs+TKIs and identified low pre-treatment platelet-to-lymphocyte ratio (PLR) values (>98.89) as an independent risk factor for a shorter median overall survival and progression-free survival.

The second most common primary malignant liver tumour is intrahepatic cholangiocarcinoma (ICC), characterised by an aggressive behaviour with only 20-40% of cases amenable of surgery at presentation, and a 5-year survival of only 30-40% after complete resection. In their multicentric analysis on a cohort of 374 patients, Zhang et al. developed a novel classification based on pre-operative inflammatory and immune status (merging together the systemic immune-inflammatory index (SII) and the albumin bilirubin (ALBI) grade) that was able to serve as a reliable prognostic indicator for postoperative overall and recurrence-free survival in patients with ICC.

Several inflammation-based scores have been proposed and evaluated, and He et al. retrospectively analysed 399 ICC patients comparing 8 different scores to determine the one with the best survival outcomes predictive value. The modified Glasgow Prognostic Score (mGPS), a combination of C-reactive protein (CRP) and albumin levels, emerged as the most sensitive, efficient, simple, rapid, and widely applicable preoperative prognostic factor for ICC patients, with elevated mGPS scores indicating a poor prognosis.

Several challenges still exist in the complex field of primary liver tumours, where clinicians face difficulties in obtaining early diagnoses and selecting the optimal treatments to grant patients the best possible outcomes. The dysregulation of the tumour microenvironment, associated with inflammation, is a well-established contributor to carcinogenesis and tumour progression. Therefore, the identification of early, reliable and validated prognostic inflammatory markers is of paramount importance in the context of an increasingly personalized-medicine approach.

## Author contributions

GR: Conceptualization, Writing – original draft. DT: Conceptualization, Writing – review & editing.

## References

[1] Reig M Forner A Rimola J Ferrer-Fabrega J Burrel M Garcia-Criado A. BCLC strategy for prognosis prediction and treatment recommendation: The 2022 update. J Hepatol. (2022) 76:681–93. doi: 10.1016/j.jhep.2021.11.018 PMC8866082 34801630

[2] Vitale A Cabibbo G Iavarone M Vigano L Pinato DJ Ponziani FR. Personalised management of patients with hepatocellular carcinoma: A multiparametric therapeutic hierarchy concept. Lancet Oncol. (2023) 24:e312–22. doi: 10.1016/S1470-2045(23)00186-9 37414020

